# Supplementary material for: Associations between patterns in comorbid diagnostic trajectories of individuals with schizophrenia and etiological factors
Source: Nat Commun. 2021 Nov 16;12:6617. doi: 10.1038/s41467-021-26903-7 (PMC8595374; doi:10.1038/s41467-021-26903-7)
Supplement: Supplementary file 1 — Supplementary Information [file 41467_2021_26903_MOESM1_ESM.pdf]

# Supplementary Information

*Morten Dybdahl Krebs, Gonçalo Espregueira Themudo, Michael Eriksen Benros, Ole Mors, Anders D. Børglum, David Hougaard, Preben Bo Mortensen, Merete Nordentoft, Michael J. Gandal, Chun Chieh Fan, Daniel H. Geschwind, Andrew J. Schork, Thomas Werge, and Wesley K. Thompson*

*2021-07-02*

This is a document providing the supplementary figures and tables for the paper "Patterns in Comorbid Diagnostic Trajectories of Individuals with Schizophrenia Associate with Etiological Factors" by Krebs et al.

R code is available at https://github.com/MortenKrebs/Trajectories_in_schizophrenia. Main functions are in the form of an R-package named `diagtract`. We use the notation `package::function` to refer to specific R functions.

# Supplementary Note 1

## Data Material

The Study was performed using the iPSYCH cohort (See Pedersen et al. [9]). The primary cohort included all individuals born in Denmark between May 1, 1981 and December 31, 2002, and diagnosed with schizophrenia before December 31, 2012 (N= 5432). An additional non-overlapping cohort of individuals born in Denmark between May 1, 1981 and December 31, 2005, and diagnosed with schizophrenia before December 31, 2016 was obtained for replication (N=870).

## Matched controls

To identify population controls with similar characteristic to the case sample, we sampled from the non-overlapping part of the (N=30 000) iPSYCH population sample, matching on age and sex.. Matching was done using propensity score matching as implemented in the `R` function `MatchIt::matchit` [5] with matching on age at end of follow up and sex settings `method="nearest"`, `distance="glm"` and `ratio=2`. We observed a standardized mean difference[1] in the covariates of 6% for gender and 0.7% for age, which we considered acceptable as they were below the typical 10% recommendation[1]. As outlined in Table 1 this resulted in a (N=10 864) control cohort with similar age and gender characteristic to the case sample."

# Supplementary Note 2

## Diagnosis State Sequences:

Using the first event of a diagnosis with one of the eight categories of psychiatric diagnoses outlined in Table S1 and increments of 12 months, comorbidity trajectories were transformed into sequences. In case of multiple comorbidities, alphabet extensions were used (For illustration, see Figure S1). Among the 5432 included subjects, the raw (unimputed) sequences have lengths ranging from 16 to 35 years. The alphabet include 193 unique, observed states (out of the 256 possible unique states).

# Supplementary Note 3 - Sequence Dissimilarities

## Transition Rates

To obtain population-wide estimates of transition probabilities, the random population cohort (See Pedersen et al. [9] for details) and estimates were computed using inverse sampling probability weighting (Bowman II). To estimate sampling probability, the distribution of age and sex in the Danish population was obtained (publicly available from Statistics Denmark). Inverse sampling probability weighting was performed taking age and sex into account. Time-varying Transition Rates were computed with `TraMineR::seqtrate()`.

## Substitution Costs

To accommodate the large sequence alphabet substitution costs were set to take into account overlap in states using Jaccard distance (for examples see Table S3) :

$$d_J(A,B) = 1 - \frac{|A \cap B|}{|A \cup B|}$$

## Right-censoring

To calculate dissimilarities between sequences with right-censoring, we used inferred states weighted by the probabilities of that state, given the last observed state in the sequence. When calculating the dissimilarity between two sequences $i$ and $j$ of unequal length, the dissimilarity, $D(i,j)$ was defined as:

$$D(i,j) = d_{obs} + d_{inf}$$

where $d_{obs}(i,j)$ is the dissimilarity between the sequences before right-censoring occurs and $d_{inf}$ is the sum of the substitution cost matrix weighted by the inferred probabilities.

## Imputation

When imputing the sequences, the last state before censoring was set to the accumulated set of diagnoses at censoring (i.e. if one or more diagnoses had occurred between the last birthday and the end of follow-up, state used for imputation was different than the last state in the observed (unimputed) sequence). To preserve this information, also in the longest sequences, all sequences were imputed up to length 36 years.

For the right censored states, probability-weighted substitution costs were used:

$$d_{inf}(i,j) = \sum_{t=1}^{t_{max}} (P(i)_t P(j)_t)^T \times C$$

where $P(i)_t$ and $P(j)_t$ are the vectors of the probabilities of sequence $i$ and $j$ being equal each state of the alphabet at time $t$. $^T$ indicate the outer product and $C$ is the substition cost matrix.

The time dependent probability vectors $P(i)_t$ are estimated assuming a first-order markov property $P(X_{t+1} = s) = P(X_{t+1} = s|X_t = s_t)$ for all states $s_1, s_2, ..., s_t, s$.

Software to perform these computations is available in `diagtraject::mis.cost()`.

# Supplementary Note 4

## Multidimensional scaling

Metric multidimensional scaling using `vegan::wcmdscale()`.

## Jackknife Stability of MDS

Jackknife stability of MDS over $n = 100$ samples of the dissimilarity matrix. Where stability is measured as the stability coefficient:

$$\mathrm{ST} = 1 - \frac{\sum_{i=1}^{n} \parallel X_i^* - \bar{X}^* \parallel^2}{\sum_{i=1}^{n} \parallel X_i^* \parallel^2}$$

where $X_i^*$ is the Jackknife MDS solutions after a Procrustes transformation and $\bar{X}^*$ is the average result across all jackknife iterations. This can be interpreted as the ratio of between and total variance (See de Leeuw and Saporta [6]). This was computed using `diagtraject::bootmds()`, highly inspired by the implementation in `smacof::bootmds`.

# Supplementary Note 5

## Clustering

Clustering was a hierarchical agglomerative clustering using Ward's method as implemented in the `stats::hclust(,method="ward.D2")`. By visual inspection, the number of clusters were selected based on the location of elbow in the $R^2$ (See Figure S 8)

## Cluster stability

Multidimensional scaling and clustering was repeated with 100 random subset samples. The overlap between the clustering of the full data-set and the bootstrap permutations was assessed using the mean Jaccard coefficient.

# Supplementary Note 6

## Sensitivity

To test the sensitivity of the associations to different parameter settings in sequence analysis, we selected a subset of subjects (N=3508) that did not require imputation. We did this by only including subjects followed for more than 25 years, and by only looking at the first 25 years of the trajectories. We then performed the sequence analysis with three different substitution cost settings:

- Constant costs
- Jaccard Distance
- 1-Simple matching coefficient (Equivalent to Multichannel Sequence Analysis)

We then computed dissimilarities using three different sequence alignment methods:

- Hamming Distance (HAM)
- Optimal Matching with indel costs of $max(SC)/2$ (OM_0.5)
- Optimal Matching with indel costs of $max(SC)$ (OM_1)

Additionally we added six state distribution based dissimilarities [2]. We then performed multidimensional Scaling and MANCOVA as described above.

In Table S11 we show the results of these analyses. The 'with imputation' column shows the results using the same dissimilarity estimates as in the main analysis but doing the multidimensional scaling and association analysis in the >25 years follow-up subsample only. Here we see that while most associations remain, the educational attainment PGS is not found, possibly related to the fact that we have relatively few samples with genotypes available in this older part of the cohort (N=1024). Next, in the OM_jacc_1 column, we use the same alignment method, substitution cost and indel cost, but look only at the part of the trajectories that are fully observed (i.e., we compare sequences with lengths of exactly 25 years). Here we see that most associations attenuate a little but that the overall pattern seems unchanged, which could be ascribed to the loss of information from censoring all individuals at age 25. Varying the indel costs have very small effects, indicating that the alignment is driven largely by substitutions. Defining the substitution costs as the simple matching coefficient rather than the jaccard distance (which is equivalent to multi-channel sequence analysis with two states per channel) also doesn't change the general picture, but a constant substitution cost (i.e. ignoring that some states consist of overlapping combinations of diagnoses and treating all as equally similar) seems to have a bigger impact, but also gives similar patterns of association and here, indels have the potential to influence the alignment more, but also does not seem to change the overall pattern much. The Euclidian distance between the probability distributions is an alternative measure, but when the number of distributions is high it approximates the hamming distance-based alignment procedures with a constant cost[11]. In accordance with this, we also found that the general patterns of association was found here. The chi2 metric puts weight on the rarity of at state when the dissimilarities are computed and have previously been shown to be fundamentally different from all other sequence analysis methods applied here[11], none of our associations were seen when the chi2 based metrics where used, probably since the associations we find were associated to differences in the sequences of common states primarily. Taken together, we find that the reported associations are robust across a range of alignment methods and parameter choices.

# Supplementary Note 7

## Note on impact of cohort effects

There are several ways in which cohort effects could be influencing the trajectories: 1) technical challenges in comparing trajectories of different length, 2) different ascertainment due to study design, 3) changing diagnostic practice, and 4) changing environment. We discuss these challenges in this note:

## Comparing trajectories of different length

When different follow-up length is present in different individuals, the standard approaches of sequences analysis can potentially be impacted. In this study we chose to perform an imputation procedure (see section ) as recommended by sequence analysis experts (e.g., Halpin [4]; Gabadinho and Ritschard [3]) to avoid this impact. A software package to conduct the analysis are provided at (https://github.com/MortenKrebs/diagtraject).

## Different ascertainment with birth year

Since the iPSYCH cohort included all individuals were born between May 1, 1981 and December 31, 2002 and diagnosed before December 31, 2012 and schizophrenia is generally diagnosed in early adulthood, we find

it fair to assume that we have a high proportion of schizophrenia cases in the older part of the sample (e.g. for those born before December 31, 1984 we have all that were diagnosed before the age of 28). However, for the younger part of the sample, the ascertainment is more incomplete (e.g. for those born after January 1, 1989, we only have those diagnosed before age 23). We illustrate the impact on the cumulative incidence of comorbidities in Figure S11. As expected, we see an over-representation of individuals with early-onset schizophrenia in individuals born after January 1, 1990 (mean age at diagnosis 18.2) compared to those born before January 1, 1990 (mean age at diagnosis 22.0), which could drive our data-driven approaches towards up-weighting disease trajectories with early onset as particularly important. However, the ascertainment will not have impact the associations with risk factors.

## Changing diagnostic practice

ICD-8 replaced ICD-10 in Denmark on Jan 1, 1994 (ICD-9 was never implemented in Denmark). Expert epidemiologists have provided ways of converting the diagnoses [10] (Table S1), though of course still limited by non-identical diagnostic criteria across the two systems. Since all individuals in iPSYCH were born after May 1, 1981, none had been diagnosed with schizophrenia under ICD-8. For other adult onset disorders the number of individuals diagnosed under ICD-8 will also be very small (e.g. we also see for mood disorders and eating that all diagnoses were assigned under ICD-10). However, some will have received child and adolescent psychiatric diagnoses under ICD-8 (For ASD, 7 of 389 were diagnosed under ICD-8 of which some (<5) also have a ICD-10 diagnosis. For ADHD , 16 of 598 were diagnosed under ICD-8 of which most (>11) also have a ICD-10 diagnosis). While the number of diagnoses assigned under ICD-8 are very small, there could still be differences in the diagnostic practice that happens more gradually after the change in diagnostic system. In Figure S12 we show the cumulative incidence at age 21 of each of the eight comorbidities stratified by birth before 1988 and between 1988 and 1992. Here we observe an increase in the incidence of mood disorders, ASD and childhood disorders, but a decrease in the incidence of personality disorder. In addition to changing diagnostic criteria, the organization of mental health services in Denmark has also changed. E.g. while the number of beds where was halved from the 1980 to 2000, number of patients treated has increased [8]. Finally, from 1995 the registration of diagnoses in the PCRR also include diagnosed from patients treated in outpatient clinics. [7]

## Changing environment

Cohorts effects could arise if environment factors related to disease have changed with time. E.g. studies have found that the composition of cannabis products has changed over time [12] .

## Effects on associations between trajectory dissimilarities and risk factors

In our sensitivity analyses (Section , Table S11 ), we selected only patients born before January 1, 1991 (i.e. with more than 25 years follow-up). We looked only at their trajectory up to age 25. In Table Table S11 we show the results of these analyses. The 'with imputation' column shows the results using the same dissimilarity estimates as in the main analysis, but doing the multidimensional scaling and association with risk factors and outcomes analyses in the >25 years follow-up subsample only. Since all individuals in this subsample were born before 1991, these analyses should be less susceptible to the possible cohort effects discussed above. Here we see that while most associations remain, the educational attainment PGS is not found, possibly related to the fact that we have relatively few samples with genotypes available in this older part of the cohort (N=1024).

# Supplementary Note 8

## Choice of replication sample

Since the study relied on the iPSYCH2012 study [9], which was defined based on diagnoses assigned before December 31, 2012, we could obtain a population representative sample using the 5432 individuals diagnosed with schizophrenia before that time point. However, because iPSYCH2012 also included cohorts of ADHD, ASD, affective disorders and a control sample, additionally 870 individuals had been diagnosed with schizophrenia before December 31, 2016. This sample was only a subset of individuals diagnosed with schizophrenia in this time period (Table S12) and due to the iPSYCH inclusion criteria, it did not have the population representative properties of the 2012 sample.

We hypothesized that this sampling could lead to false positives due to ascertainment bias if these individuals were included in the discovery analysis (Figure S 13), but that including them as a replication cohort, would be less sensitive to this effect.

## Theoretical example

To understand this better, we considered the simple scenario that in a population $P$ of size $N$ with two uncorrelated variables $x$ and $y$, two samples, $A$ and $B$, have been drawn. Sample $A$ ($\mathrm{SCZ}_{2012}$) is a random sample of size $n_a << N$, but $B$ ($\mathrm{SCZ}_{2016}$) is dependent on $x$ and $y$ and has size $n_b << N$. This will lead to dependence of $x$ and $y$ in sample $B$, which is often referred to as Berkson's bias.

Our hypothesis was that while merging samples $A$ and $B$ and splitting them randomly into two, one used for discovery, and one for replication, could lead to type I error due to Berkson's bias — using $A$ as discorvery and $B$ and replication would not lead to type I errors.

## Simulations

Setting $N = 10000$, we simulated two independent normal random variables $x$ and $y$ and defined $A$ as a random subset of $P$ while $B$ was defined as the subset of $P$ in which $x > 1$ or $y > 1$ which was not part of $A$. We compared the approach of treating $A$ and $B$ separately to merging $A$ and $B$ and spitting them randomly. Using each split, we tested for a linear relationship between $x$ and $y$ by fitting simple linear regression in each the two sets and considered p-values smaller than 0.05 in both regressions a false positive. Repeating the procedure 1000 times we estimated a false positive rate of 1 for the random split and 0.045 when $A$ and $B$ were analyses separately (See R code below).

## Interpretation

This simple simulation shows that if ascertainment bias is suspected, but a subset of the data are known to be free from this bias (e.g. a random sample of the population), analysing this subset separately and only considering findings significant in this subset adequately controls the false positive rate.

## R code

```
#Simple simulation comparing a split into population representative
# and non-representative to a half half spit under ascertainment bias

set.seed(123) # Reproducibility
false_positives <- sapply(1:1000, function(x){
n <- 10000 # Sample Size
risk_factor <- rnorm(n)
clinical_presentation <- rnorm(n)
in_study2012 <- rbinom(n, size = 1, prob = 0.15)
in_study2016 <- ((risk_factor > 1) | (clinical_presentation > 1))
in_study2016[in_study2012==1] <- 0

# our method
lm_2012 <- lm(clinical_presentation~risk_factor,subset=(in_study2012==1))
lm_2016 <- lm(clinical_presentation~risk_factor,subset=(in_study2016==1))


# splitting randomly
in_any <- ((in_study2012==1) | (in_study2016==1) )
in_first_half <- in_second_half <- in_any
in_first_half[(floor(sum(in_any)/2)+1):n] <- 0
in_second_half[1:floor(sum(in_any)/2)] <- 0
lm_a <- lm(clinical_presentation~risk_factor,subset=(in_first_half==1))
summary(lm_a)
lm_b <- lm(clinical_presentation~risk_factor,subset=(in_second_half==1))
summary(lm_b)

c(our_split=all(summary(lm_2012)$coefficient[2,4]<0.05,
  summary(lm_2016)$coefficient[2,4]<0.05), half_half=
all(summary(lm_a)$coefficient[2,4]<0.05,
  summary(lm_b)$coefficient[2,4]<0.05))

})
# false positive rate:
rowSums(false_positives)/1000
> our_split half_half
    0.045     1.000
```

Figure S 1: Most frequent sequences of psychiatric comorbidity diagnoses among the schizophrenia patients with 25 years follow-up

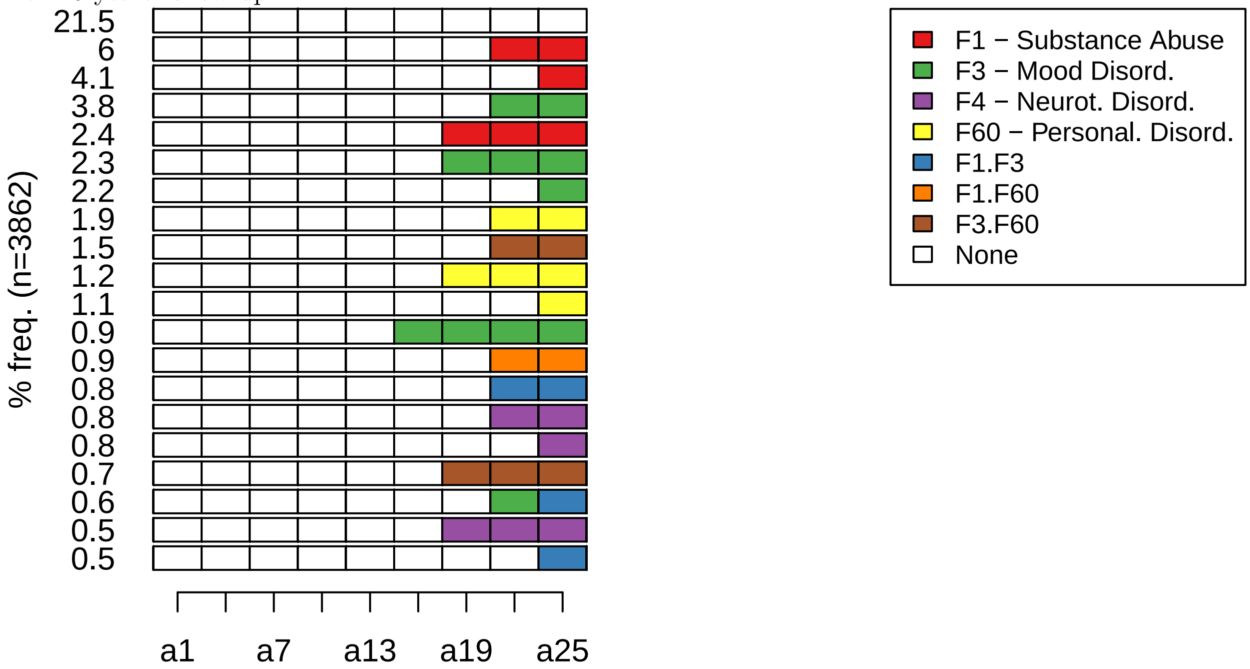


The figure displays the 20 most frequent sequences of psychiatric diagnoses (as defined in Table S1) in the first 25 years from birth for 3862 individuals born 1981-1991 and diagnosed with schizophrenia (ICD8: 295.x9 excl. 295.79; ICD10: F20.0-F20.9) before 2013. To increase representativeness increments are set to three years opposed to one year increments all analyses.

Figure S 2: Cumulative $R^2$ of multidimensional scaling with increasing number of dimensions

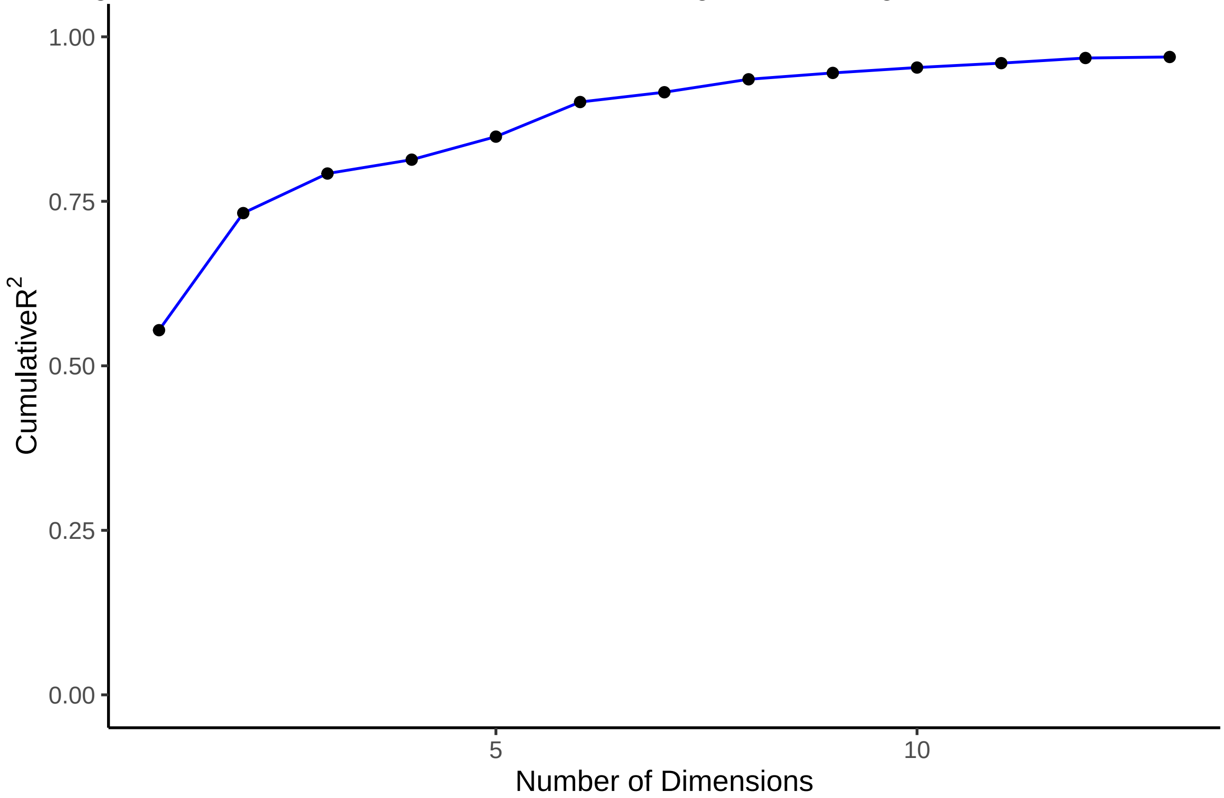


The figure displays the proportion of variance ($R^2$) in the sequence dissimilarities explained by the multidimensional scaling with increasing number of dimensions.

Figure S 3: Stressplots for multidimensional scaling solutions with increasing number of dimensions (k=2-13).

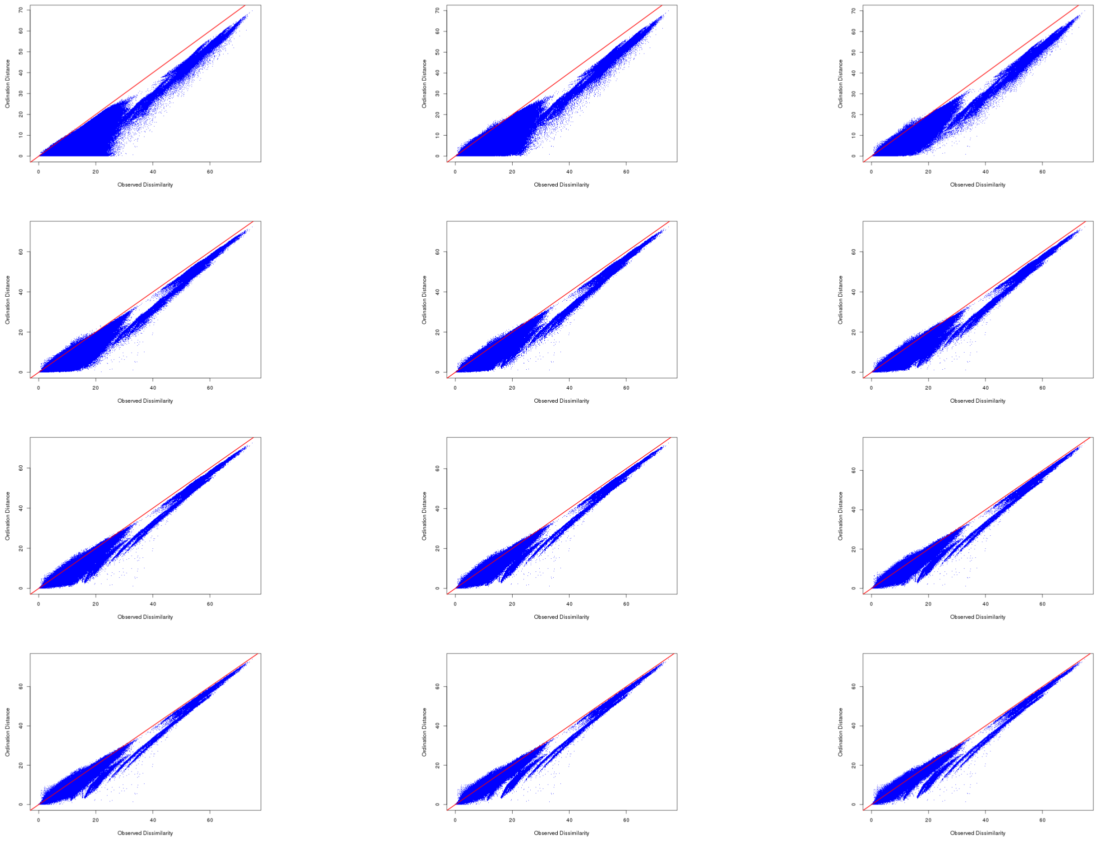


Each of the 12 figures shows the relationship between the pairwise dissimilarities of comorbidity trajectories in schizophrenia patients estimated by Sequence Analysis and the ordination distance obtained from the multidimensional scaling. Upper left is the 2-dimensional solutions the following figures show the relationship when additional dimension are added up to 13 dimensions.

Figure S 4: Prevalence and age of onset of each of the diagnoses at different quantiles of each of the MDS dimensions 1-7

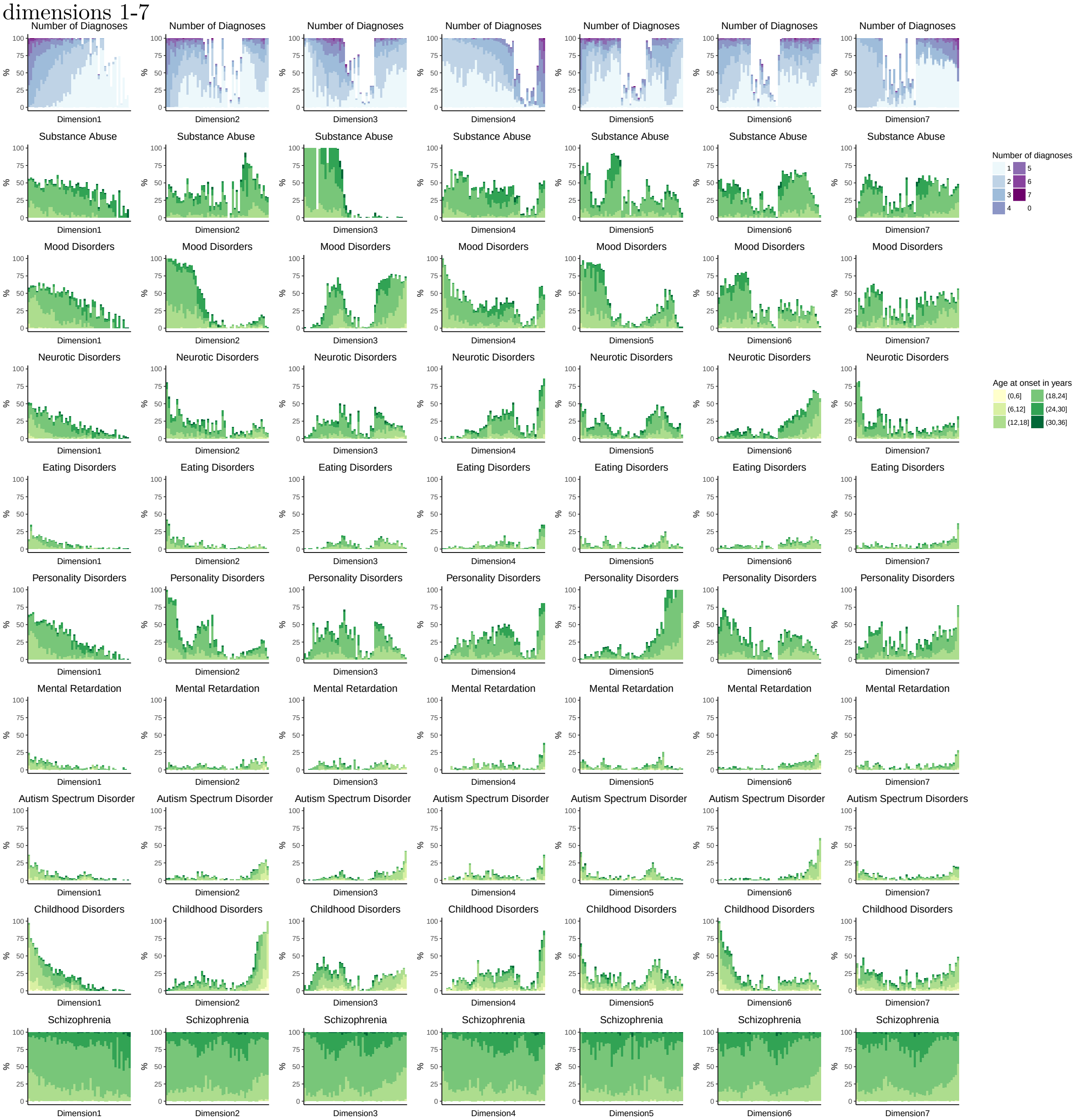


The first row displays the prevalence of different number of different categories of diagnoses (other than schizophrenia) as defined in Table S 1 in each 2-percent quantile of each of dimensions 1-7. Rows 2-9 display the frequency of each of the categories of psychiatric diagnoses with different age on onset in each 2-percent quantile of each of dimensions 1-7. Row 9 displays the frequency of different onset ages of schizophrenia for each 2-percent quantile of each of dimensions 1-7.

Figure S 5: Association of first principal MDS dimension with number of cooccurring comorbid diagnoses

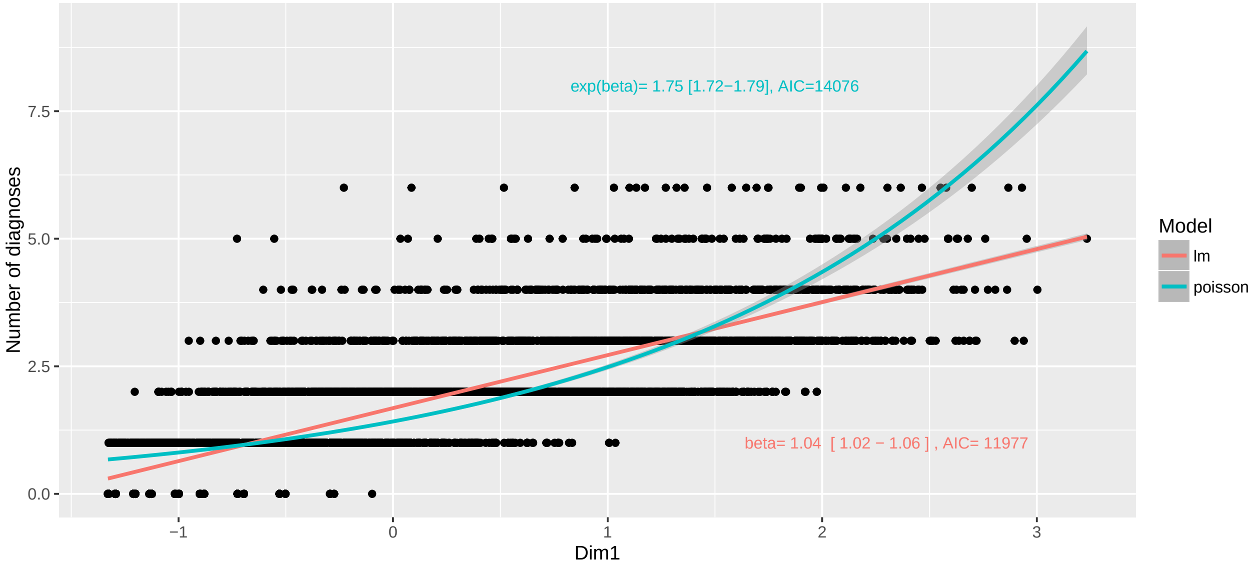


The figure displays the relationship between the value of the first principal MDS dimension of the comorbidity trajectories of the 5432 schizophrenia patients and the observed number of co-occurring psychiatric diagnoses (as defined in Table S1). The lines display the result of a linear model and a Poisson regression of the relationship.

Figure S 6: Association of the second principal MDS dimension, mood disorders and childhood disorders

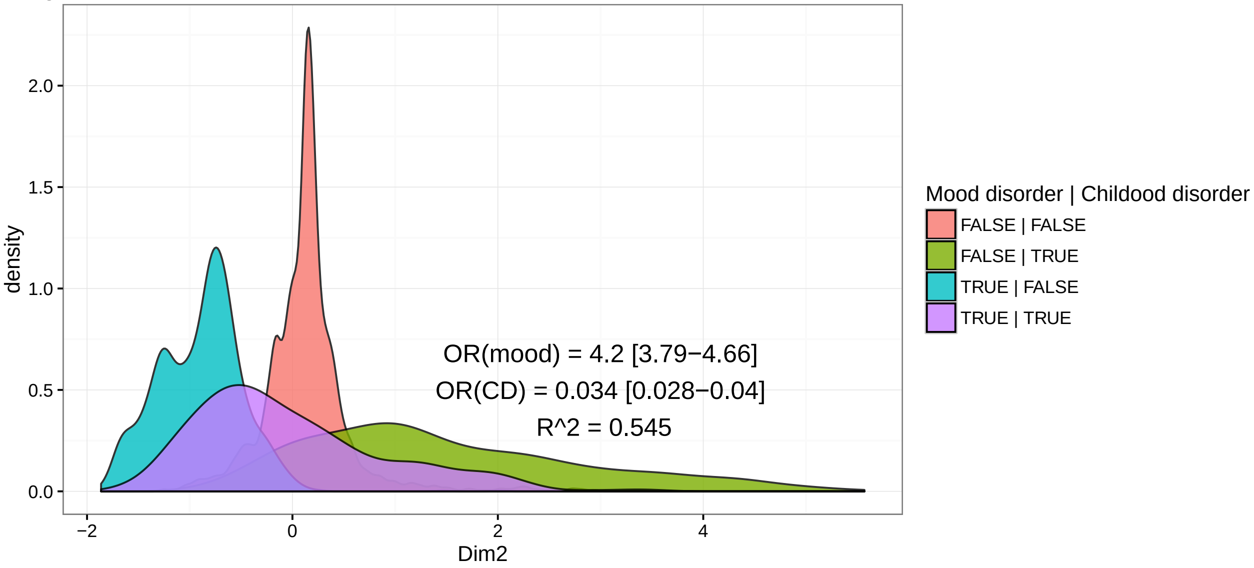


The figure displays the distribution of the value of the second principal MDS dimension of the comorbidity trajectories among the (N= 5432) schizophrenia patients stratified by the presence of mood disorders and childhood disorders (as defined in Table S1). OR(mood) and OR(CC) indicate the odds ratio of a diagnosis with mood disorders and childhood disorders respectively per standard deviation increase in the value of the second principal MDS dimension with 95% confidence intervals estimated by logistic regression. $R^2$ is the proportion of variance in the value of the second principal MDS dimension explained by this two factors estimated by linear regression.

Figure S 7: Association of the third principal MDS dimension and substance abuse

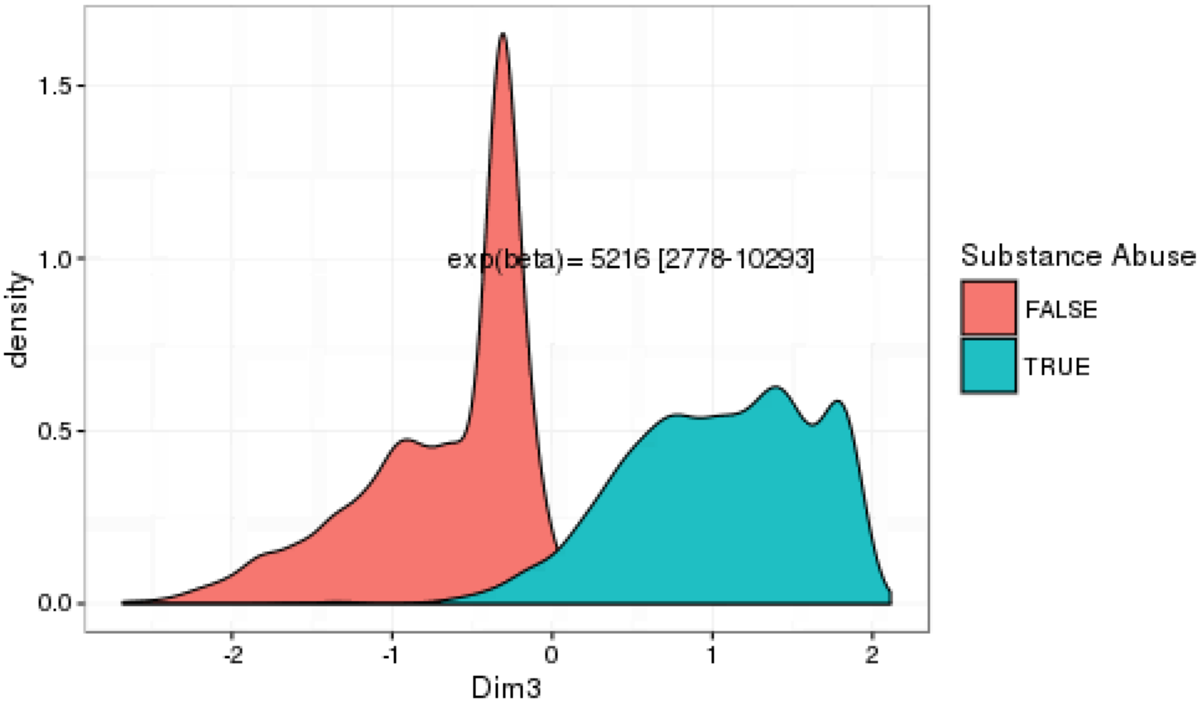


The figure displays the distribution of the value of the third principal MDS dimension of the comorbidity trajectories among the (N= 5432) schizophrenia patients stratified by the presence of substance abuse disorders (as defined in Table S1). exp(beta) indicates the odds ratio of a diagnosis with substance abuse disorders per standard deviation increase in the value of the third principal MDS dimension with 95% confidence intervals estimated by logistic regression.

Figure S 8: Proportion of variance in the values of the three principal MDS dimensions explained by clustering with increasing number of clusters

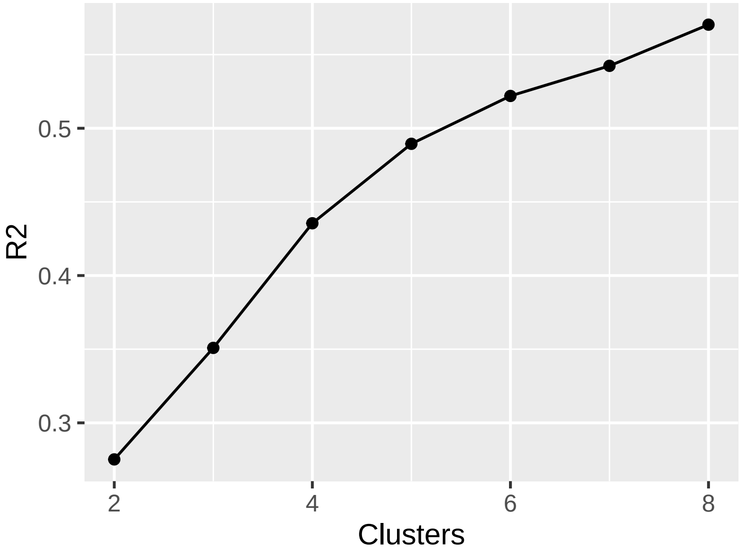


The figure displays the proportion of variance ($R^2$) in the three principal MDS dimensions of the psychiatric comorbidity sequences dissimilarities of 5432 patients with schizophrenia explained by hierarchical agglomerative clustering with number of clusters ($k$) from 2 to 8.

Figure S 9: Clustering of schizophrenia patients based on the three principal MDS dimensions of psychiatric comorbidity sequences and assessment of clustering stability under subsetting

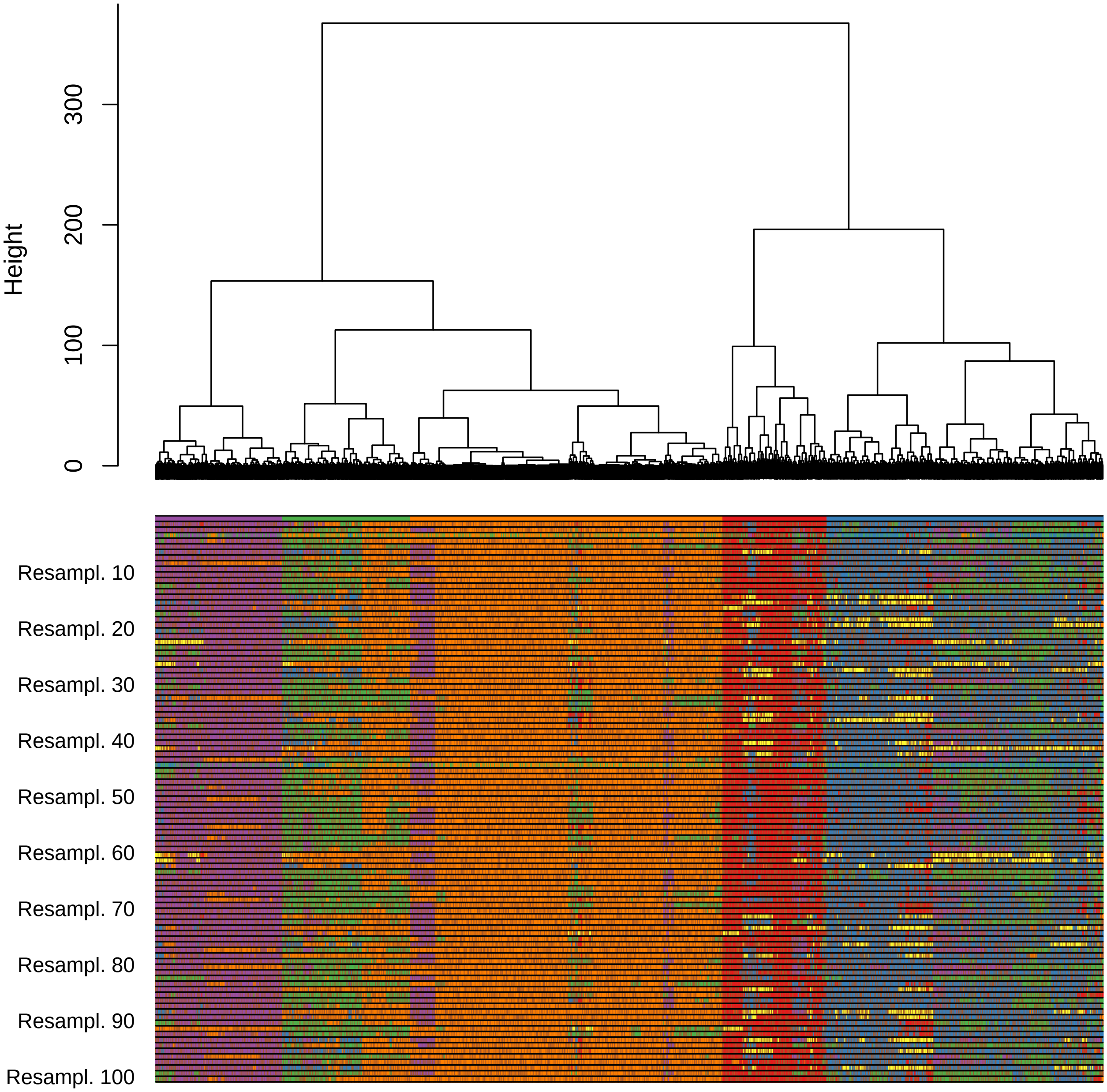


**Above:** Dendrogram of the hierarchical agglomerative clustering. **Below:** The first line is the result of the multidimensional scaling and clustering when all 5432 schizophrenia patients are included. Clusters are coloured as in Figure 4 in the main text. Lines 2-101 are the results of MDS and clustering repeated on 100 random subsets (N=3368) of the 5432 schizophrenia patients. For each permutation k=3 multidimensional scaling was computed, and subsequently a $k = 5$ hierarchical agglomerative clustering was performed. If a cluster in the subset has a significant overlap with any of the clusters in the full data set instantiated by a p-value $< 0.05$ in Fisher's exact test, all individuals in the subset cluster is given the colour of full data cluster. Yellow indicates a cluster with no significant overlap. Grey indicated individuals not included in the subset.

Figure S 10: Distribution of seven polygenic scores among individuals with schizophrenia

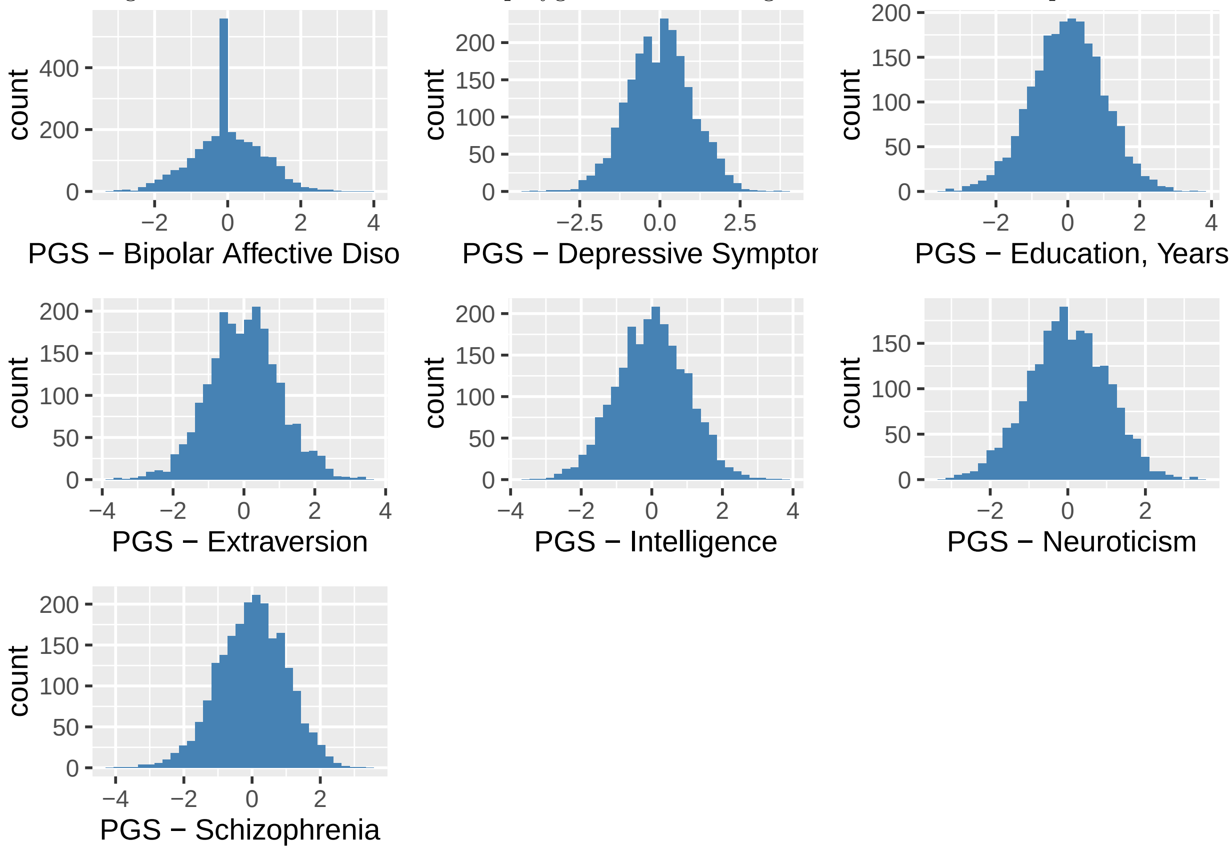


The figure displays the distribution of the centered and scaled polgenic risk score computed from summary statistics of seven published genome-wide association studies ( Table S 5) computed using LDpred ( Table S 6) for the 2147 individuals with schizophrenia genotyped on the PsychArray and passing QC.

Figure S 11: Cumulative incidence at age 23 of comorbidities diagnosed before and after schizophrenia stratified on birth before and after January 1, 1990.

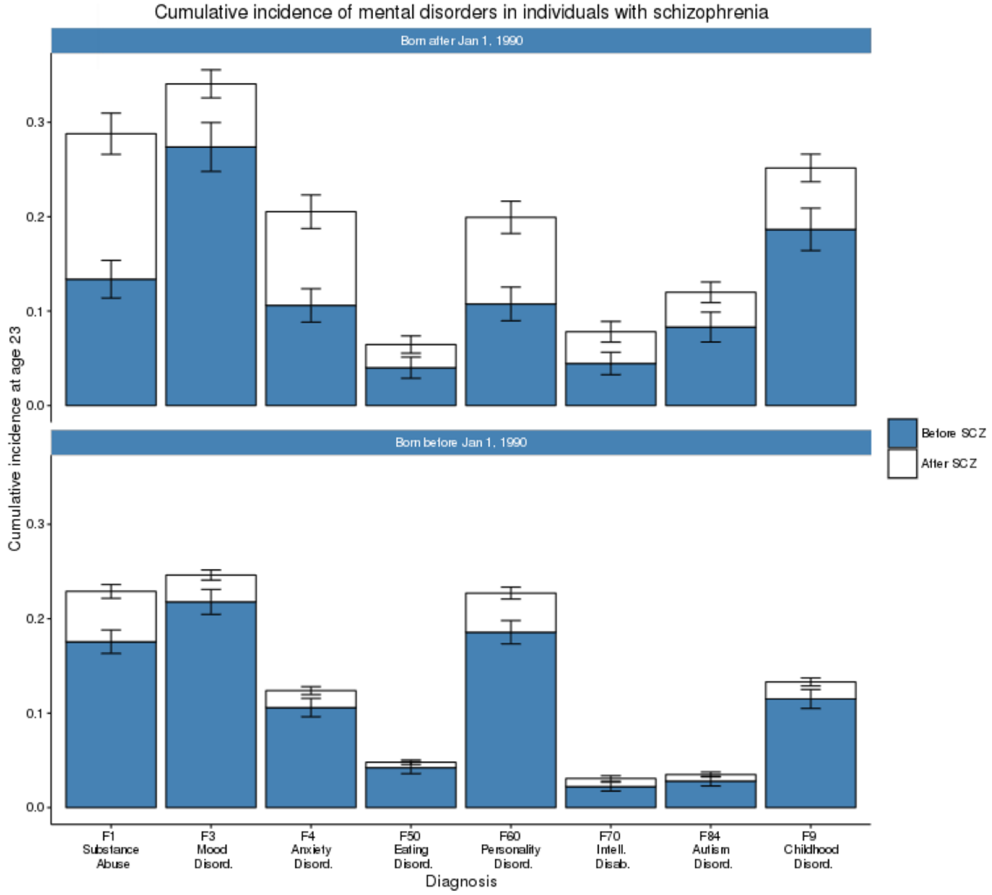


The figure shows the cumulative incidence (CI) at age 23 of each of eight categories of comorbidities in individuals with schizophrenia born after Jan 1, 1990 (above) and before Jan 1, 1990 (below). In blue are the CI before schizophrenia, in white are CI after schizophrenia. Error bars are 95%-confidence intervals. Sample sizes in the before Jan 1, 1990 group are ($N_{none}/N_{before}/N_{after}$) F1: 2500/874/601; F3: 2667/1133/323; F4: 3341/548/308; F50: 3961/194/73; F60: 2883/911/394; F70: 4021/ 77/ 104; F84: 4012/ 88/ 127; F9 3427/ 543/ 232. Sample sizes in the after Jan 1, 1988 group are ($N_{none}/N_{before}/N_{after}$): F1: 811/149/177; F3: 753/314/87; F4: 925/123/120; F50: 1101/47/28; F60: 940/125/110; F70: 1078/52/37; F84: 1031/97/42; F9: 867/216/77.

Figure S 12: Cumulative incidence of at age 21 of comorbidities diagnosed before and after schizophrenia stratified on birth before January 1, 1988 and between January 1, 1988 and January 1, 1992.

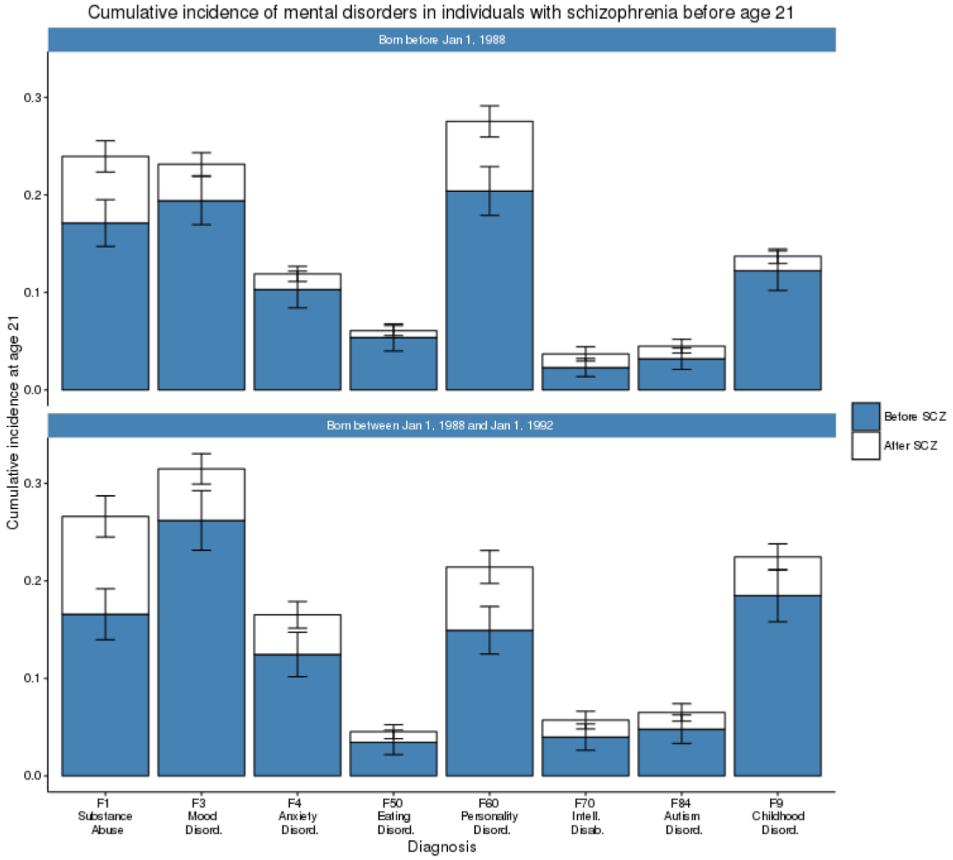


The figure shows the cumulative incidence (CI) at age 21 of each of eight categories of comorbidities in individuals with schizophrenia born before Jan 1, 1988 (above) and between Jan 1, 1988 and Jan 1, 1992 (below). In blue are the CI before schizophrenia, in white are CI after schizophrenia. Error bars are 95%-confidence intervals. Sample sizes in the before Jan 1, 1988 group are ($N_{none}/N_{before}/N_{after}$) F1: 617/163/172; F3: 681/192/116; F4: 798/103/99; F50: 916/54/33; F60: 643/203/149; F70: 956/ 23/ 25; F84: 933/ 32/ 36; F9 822/ 123/ 61. Sample sizes in the after Jan 1, 1988 group are ($N_{none}/N_{before}/N_{after}$): F1: 517/129/132; F3: 509/208/77; F4: 641/101/69; F50: 769/28/18; F60: 609/122/86; F70: 752/32/20; F84: 757/39/18; F9: 604/149/53.

Figure S 13: Schematic of the possible ascertainment issue in the 2016 sample

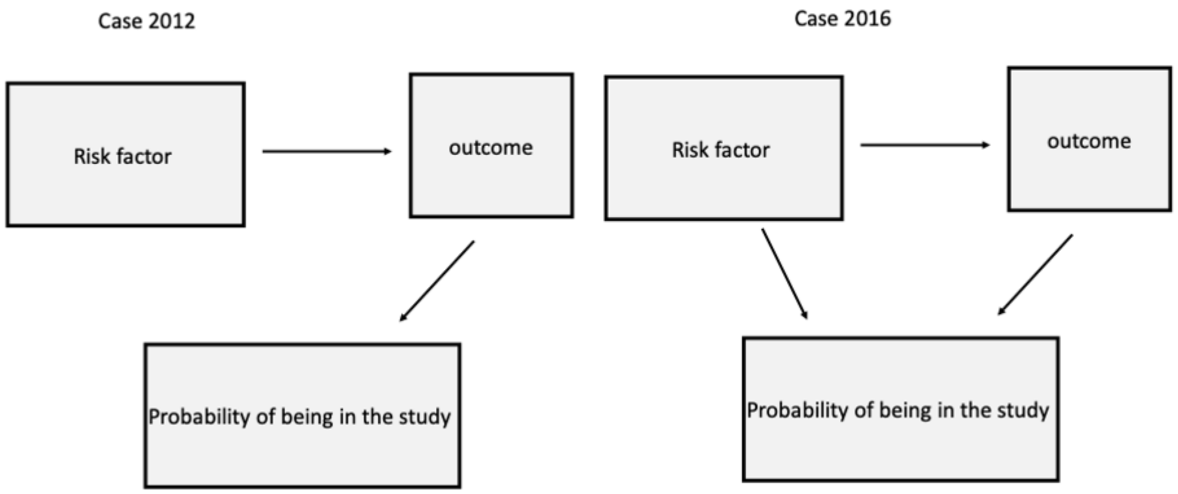

Figure S 14: Decision tree for classification of individuals without access to Danish register data

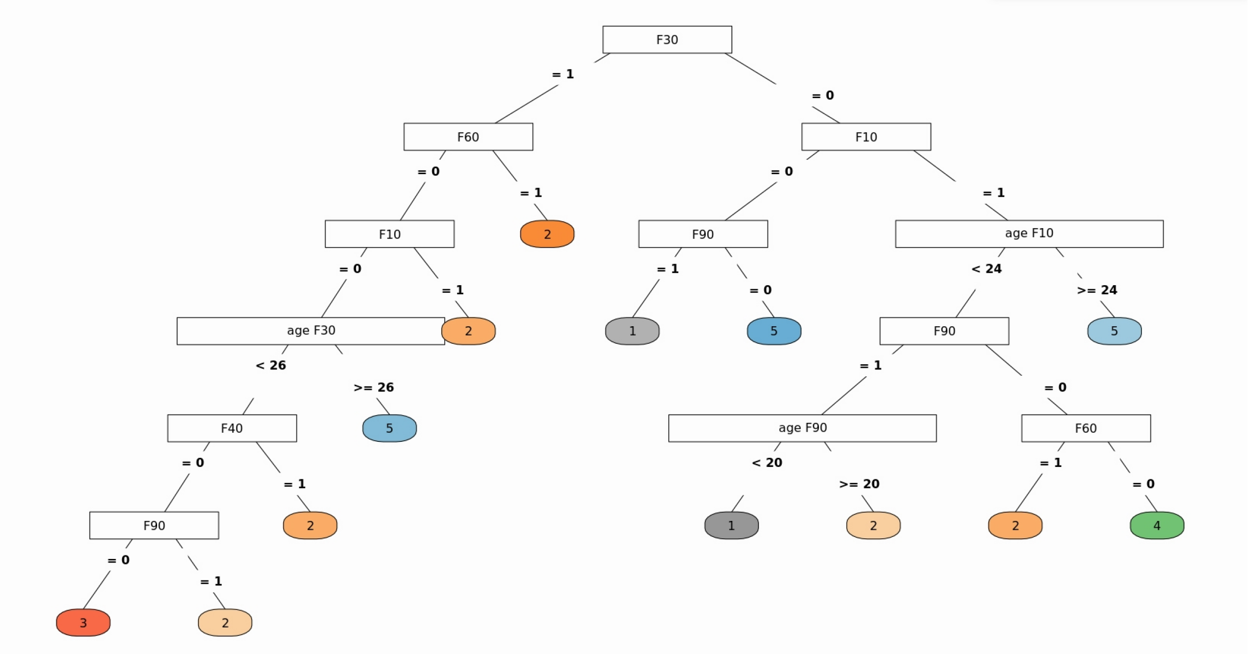


A decision tree was trained to classify patients into the groups described by the k=5 clustering based on the presence/absence of each of the eight categories of diagnoses (Table S1) and on their age at onset. This was done using the rpart software (https://cran.r-project.org/web/packages/rpart/) treating the clustering as categorical outcome (method='class'). The resulting tree presented above.

Figure S 15: Associations with risk factors and outcomes for the $k = 5$ clustering and the classification based on the decision tree presented in Figure S14 estimated by multinomial logistic regression

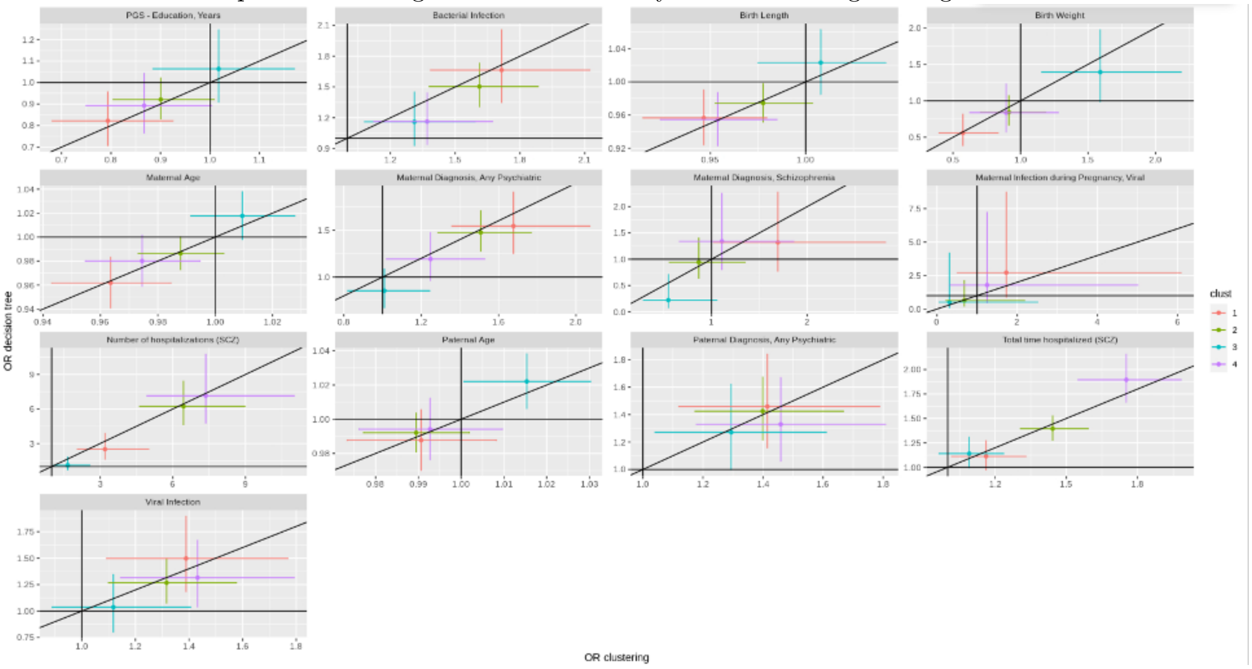


Multinomial logistic regression is conducted using either MDS-based cluster membership (x-axis) or decision tree classification (y-axis) as dependent variable sequentially using the predictor variables as independent variable and treating Cluster 5 as reference. All regressions are adjusted for age and sex. PGS - Educational attainment is adjusted additionally for 10 principal components and genotype wave. Error bars indicate 95% confidence intervals.

Table S 1: Comorbidity definitions

| Diagnosis | ICD.10 | ICD.8 |
|---|---|---|
| Substance abuse | F10-F19 | 291.x9, 294.39, 303.x9, 303.20, 303.28, 303.90, 304.x9 |
| Mood disorders | F30-39 | 296.x9 (excl 296.89), 298.09, 298.19, 300.49, 301.19 |
| Anxiety disorders and Obsessive compulsive disorder | F40.0-F40.2 F41.0-F41.1 F42 F43.0-F43.1 | 300.39 |
| Eating disorders | F50 | 305.60, 306.50, 306.58, 306.59 |
| Personality disorder | F60 | 305.60, 306.50, 306.58, 306.59 |
| Mental Retardation | F70-79 | 301.x9 (excl 301.19), 301.80, 301.81, 301.82, 301.84 |
| Pervasive developmental disorders | F84 | 299.00, 299.01, 299.02, 299.03 |
| Behavioural and emotional disorders with onset usually occurring in childhood and adolescence | F90-98 | 306.x9 308.0x |

Table S 2: Number of individuals in each group in Cox regressions of psychiatric comorbidities among the 5432 individuals with schizophrenia

| D1 | D2 | None (N) | D1 first (N) | D2 first (N) | D2 after D1 (N) | HR | 95%-conf. | p-value |
|---|---|---|---|---|---|---|---|---|
| F1 | F3 | 2007 | 1718 | 1413 | 218 | 1.04 | (0.93-1.17) | 0.47 |
| F1 | F4 | 2585 | 915 | 1616 | 196 | 0.89 | (0.76-1.03) | 0.12 |
| F1 | F50 | 3041 | 336 | 1903 | 25 | 1.00 | (0.78-1.28) | 0.98 |
| F1 | F60 | 2395 | 1240 | 1352 | 256 | 1.34 | (1.18-1.51) | $4.4 \times 10^{-6}$ |
| F1 | F70 | 3118 | 251 | 1798 | 56 | 0.82 | (0.64-1.05) | 0.12 |
| F1 | F84 | 3016 | 347 | 1832 | 25 | 0.40 | (0.31-0.53) | $3.5 \times 10^{-11}$ |
| F1 | F9 | 2768 | 826 | 1389 | 189 | 1.28 | (1.14-1.45) | $6.2 \times 10^{-}5$ |
| F3 | F1 | 2007 | 1631 | 1304 | 414 | 0.71 | (0.61-0.82) | $7.0 \times 10^{-6}$ |
| F3 | F4 | 2823 | 764 | 1378 | 279 | 1.01 | (0.85-1.19) | 0.95 |
| F3 | F50 | 3278 | 226 | 1616 | 88 | 1.38 | (1.11-1.71) | 0.0035 |
| F3 | F60 | 2598 | 1032 | 1098 | 383 | 0.85 | (0.74-0.99) | 0.036 |
| F3 | F70 | 3189 | 258 | 1603 | 50 | 0.42 | (0.29-0.60) | $2.77 \times 10^{-6}$ |
| F3 | F84 | 3171 | 291 | 1554 | 68 | 0.58 | (0.43-0.78) | $3.0 \times 10^{-4}$ |
| F3 | F9 | 2679 | 903 | 1343 | 132 | 0.69 | (0.59-0.81) | $2.6 \times 10^{-6}$ |
| F4 | F1 | 2585 | 1885 | 726 | 190 | 1.08 | (0.91-1.28) | 0.36 |
| F4 | F3 | 2823 | 1727 | 565 | 159 | 1.48 | (1.27-1.71) | $2.4 \times 10^{-7}$ |
| F4 | F50 | 4038 | 297 | 884 | 37 | 1.52 | (1.17-1.97) | 0.0016 |
| F4 | F60 | 3179 | 1294 | 539 | 179 | 1.57 | (1.34-1.85) | $3.8 \times 10^{-8}$ |
| F4 | F70 | 4023 | 281 | 847 | 33 | 1.05 | (0.76-1.45) | 0.76 |
| F4 | F84 | 3979 | 321 | 836 | 36 | 0.90 | (0.66-1.24) | 0.52 |
| F4 | F9 | 3456 | 947 | 648 | 89 | 1.20 | (1.02-1.43) | 0.032 |
| F50 | F1 | 3041 | 2046 | 270 | 67 | 0.80 | (0.52-1.22) | 0.29 |
| F50 | F3 | 3278 | 1877 | 139 | 89 | 1.89 | (1.44-2.48) | $5.1 \times 10^{-6}$ |
| F50 | F4 | 4038 | 1068 | 196 | 63 | 1.43 | (1.00-2.03) | 0.049 |
| F50 | F60 | 3651 | 1476 | 137 | 86 | 1.35 | (1.00-1.83) | 0.052 |
| F50 | F70 | 4752 | 319 | 268 | 9 | 0.86 | (0.44-1.67) | 0.65 |
| F50 | F84 | 4704 | 375 | 259 | 7 | 1.56 | (0.91-2.68) | 0.11 |
| F50 | F9 | 4009 | 1089 | 217 | 26 | 0.92 | (0.65-1.31) | 0.64 |
| F60 | F1 | 2395 | 1712 | 916 | 338 | 1.77 | (1.53-2.05) | $3.0 \times 10^{-14}$ |
| F60 | F3 | 2598 | 1656 | 774 | 218 | 2.04 | (1.80-2.32) | $2.0 \times 10^{-28}$ |
| F60 | F4 | 3179 | 844 | 943 | 213 | 1.92 | (1.64-2.26) | $1.5 \times 10^{-15}$ |
| F60 | F50 | 3651 | 261 | 1192 | 60 | 1.61 | (1.29-2.01) | $2.2 \times 10^{-5}$ |
| F60 | F70 | 3603 | 272 | 1238 | 41 | 1.04 | (0.77-1.40) | 0.81 |
| F60 | F84 | 3521 | 337 | 1260 | 32 | 0.71 | (0.51-0.99) | 0.042 |
| F60 | F9 | 3143 | 932 | 933 | 1150 | 1.92 | (1.67-2.21) | $5.9 \times 10^{-20}$ |
| F70 | F1 | 3118 | 2038 | 193 | 63 | 1.79 | (1.29-2.49) | 0.00049 |
| F70 | F3 | 3189 | 1967 | 214 | 30 | 1.01 | (0.73-1.40) | 0.93 |
| F70 | F4 | 4023 | 1114 | 216 | 39 | 1.51 | (1.04-2.20) | 0.030 |
| F70 | F50 | 4752 | 357 | 272 | 9 | 0.91 | (0.46-1.78) | 0.77 |
| F70 | F60 | 3603 | 1539 | 194 | 44 | 1.33 | (0.93-1.89) | 0.11 |
| F70 | F84 | 4764 | 353 | 233 | 17 | 1.36 | (0.82-2.23) | 0.23 |
| F70 | F9 | 4087 | 1078 | 172 | 20 | 2.48 | (1.90-3.24) | $2.9 \times 10^{-11}$ |
| F84 | F1 | 3016 | 2060 | 295 | 55 | 0.50 | (0.32-0.77) | 0.0019 |
| F84 | F3 | 3171 | 1947 | 244 | 46 | 2.18 | (1.62-2.93) | $3.1 \times 10^{-7}$ |
| F84 | F4 | 3979 | 1108 | 267 | 40 | 2.17 | (1.51-3.13) | $3.0 \times 10^{-5}$ |
| F84 | F50 | 4704 | 349 | 324 | 14 | 1.11 | (0.52-2.38) | 0.78 |
| F84 | F60 | 3521 | 1562 | 267 | 37 | 1.38 | (0.93-2.05) | 0.11 |
| F84 | F70 | 4764 | 298 | 295 | 17 | 2.09 | (1.27-3.44) | 0.0035 |
| F84 | F9 | 4057 | 1056 | 196 | 41 | 2.18 | (1.66-2.87) | $2.0 \times 10^{-8}$ |
| F9 | F1 | 2768 | 1749 | 543 | 313 | 3.03 | (2.48-3.69) | $5.2 \times 10^{-28}$ |
| F9 | F3 | 2679 | 1780 | 694 | 185 | 1.17 | (0.95-1.42) | 0.13 |
| F9 | F4 | 3456 | 943 | 741 | 161 | 1.90 | (1.51-2.39) | $4.1 \times 10^{-8}$ |
| F9 | F50 | 4009 | 312 | 939 | 36 | 1.24 | (0.84-1.85) | 0.28 |
| F9 | F60 | 3143 | 1276 | 601 | 248 | 1.82 | (1.47-2.26) | $4.3 \times 10^{-8}$ |
| F9 | F70 | 4087 | 234 | 861 | 76 | 1.01 | (0.65-1.57) | 0.97 |
| F9 | F84 | 4057 | 279 | 829 | 69 | 1.45 | (1.05-1.98) | 0.02 |

D1 first diagnosis, D2 second diagnosis, HR hazard ratio, 95%-conf. 95%-confidence interval. p-values are from a two-sided Wald test with one degree of freedom. p-values are unadjusted

Table S 3: Substitution cost for the first five states in the alphabet

| | censored | F1 | F1.F3 | F1.F3.F4 | F1.F3.F4.F50 |
|---|---|---|---|---|---|
| censored-> | 0 | 0 | 0 | 0 | 0 |
| F1-> | 0 | 0 | 0.5 | 0.66 | 0.75 |
| F1.F3-> | 0 | 0.5 | 0 | 0.33 | 0.5 |
| F1.F3.F4-> | 0 | 0.66 | 0.33 | 0 | 0.25 |
| F1.F3.F4.F50-> | 0 | 0.75 | 0.5 | 0.25 | 0 |

Table S 4: Cluster Stability estimated by the mean jaccard overlap between the clustering of the full data and 100 random subsets

| Cluster | N | Jaccard |
|---|---|---|
| 1 | 597 | 0.6552152 |
| 2 | 1580 | 0.5962693 |
| 3 | 734 | 0.3458341 |
| 4 | 729 | 0.6499494 |
| 5 | 1792 | 0.7256697 |

MDS and clustering was repeated in 100 random subset (size 2/3) of the sequences.
The overlap between the clustering of the full dataset and the bootstrap permutations was assessed using the mean jaccard coefficient.

Table S 5: Summary Statistics used for Polygenic Score Calculations

| Phenotype | $N_{cases}$ | $N_{controls}$ | PMID | |
|---|---|---|---|---|
| Bipolar Affective Disorder | 9412 | 137760 | 173062 | Biorxiv |
| Depressive Symptoms | 161460 | NA | 27089181 | |
| Education, Years | 293723 | NA | 27225129 | |
| Extraversion | 160713 | NA | 24828478 | |
| Performance in intelligence tests | 269867 | NA | 29942086 | |
| Neuroticism | 170911 | NA | 27089181 | |
| Schizophrenia | 36989 | 113075 | 25056061 | |

$N_{cases}$ indicate number of cases in the discovery sample or in total for quantitative traits

Table S 6: Association of the seven polygenic risk scores with Schizophrenia

| Phenotype | p* | $R^{2*}$ |
|---|---|---|
| **Bipolar Affective Disorder** | **4.35e-18** | **0.0069** |
| **Depressive Symptoms** | **9.66e-22** | **0.0084** |
| **Education, Years** | **3.89e-05** | **0.0015** |
| Extraversion | 9.90e-03 | 0.0006 |
| Performance in intelligence tests | 4.30e-01 | 0.0001 |
| **Neuroticism** | **2.61e-23** | **0.0091** |
| **Schizophrenia** | **4.73e-39** | **0.0158** |

Polygenic Scores were computed for Chromosomes 1-22 using LDpred. All SNPs with an info score >0.8 and MAF >0.05 were included. No p-value thresholding was used and LDradius was set to 200.
* Based on a two-sided one degree of freedom Wald test in a binomial logistic regression of 2861 cases and 18843 controls, adjusting for age, sex,10 principal components of genetic similarity and genotyping wave.
Pseudo$R^2$ is calculated using Nagelkerke's method - comparing the full model to the model without the PGS.
P-values printed in bold indicates p<0.0001. p-values are unadjusted.

Table S 7: Schizophrenia risk factors and outcomes obtained from national registries

| Variable | Value | N |
|---|---|---|
| APGAR-5[ab] | 0-6 | 52 |
| | 7-9 | 340 |
| | 10 | 4978 |
| Birth Length (cm)[a] | <40 | 30 |
| | 41-46 | 163 |
| | 47-50 | 1578 |
| | 51-55 | 3294 |
| | >56 | 250 |
| Gestational Age[a] | <30 | 43 |
| | 30-34 | 72 |
| | 34-37 | 327 |
| | 37-42 | 3825 |
| | >42 | 44 |
| Birth Weight Score[ac] | 0-20 | 935 |
| | 20-40 | 868 |
| | 40-60 | 849 |
| | 60-80 | 794 |
| | 80-100 | 851 |
| Maternal Smoking in Pregnancy[ad] | Unknown | 4342 |
| | No | 565 |
| | Yes | 525 |
| Maternal Infection during Pregnancy, Viral[e] | No | 5412 |
| | Yes | 20 |
| Maternal Infection during Pregnancy, Bacterial[e] | No | 5270 |
| | Yes | 162 |
| Viral Infection | no | 4442 |
| | yes | 990 |
| Bacterial Infection | no | 3905 |
| | yes | 1527 |
| CNS Infection | no | 5378 |
| | yes | 54 |
| Otitis Infection | no | 4940 |
| | yes | 492 |
| | no | 5232 |
| | yes | 114 |

| | | |
|---|---|---|
| Paternal Diagnosis, Schizophrenia[e] | | |
| | Unknown | 86 |
| Paternal Diagnosis, Any Psychiatric[e] | no | 4251 |
| | yes | 1095 |
| | Unknown | 86 |
| Maternal Diagnosis, Schizophrenia[e] | no | 5210 |
| | yes | 142 |
| | Unknown | 80 |
| Maternal Diagnosis, Any Psychiatric[e] | no | 3980 |
| | yes | 1372 |
| | Unknown | 80 |
| Maternal Age[e] | <20 | 448 |
| | 20-27 | 2104 |
| | 27-32 | 1182 |
| | 32-40 | 616 |
| | >40 | 33 |
| Paternal Age[e] | <20 | 147 |
| | 20-27 | 1444 |
| | 27-32 | 1384 |
| | 32-40 | 1088 |
| | >40 | 261 |
| Number of hospitalizations (SCZ)[f] | 0-1 | 4212 |
| | 1-2 | 607 |
| | 2-3 | 230 |
| | 3-4 | 141 |
| | >4 | 242 |
| Total time hospitalized (SCZ)[g] | 0-1 | 2289 |
| | 1-30 | 2130 |
| | 30-90 | 800 |
| | 90-365 | 213 |

[a] Data obtained from the Medical Birth Register (MBR)

[b] APGAR-5: Appearance, Pulse, Grimace, Activity, Respiration at 5 minutes

[c] When calculating the value of birth weight score all singletons in the MBR born between 1981 and 2005 and having information of birth weight as well as gestational age were divided according to sex, gestational age in weeks, and for persons with gestational age of 28 weeks or more also into the following groups of calendar year at date of birth: 1981-1985, 1986-1990, 1991-1995, 1996-2000, 2001-2005. The variable birth weight score contains the proportion of persons with same sex, gestational age, and calendar period (only if gestational age is 28 weeks or larger) who have the same or a smaller birth weight than the index person.

[d] Maternal smoking was not in the register before 1991., From 1991 to 1996 it was registered as a binary (smoker of non-smoker). In the period 1997 to 2005 the register had more refined data, but this was transformed in a binary (non-smoker=non-smoker. smoker= smoker, stopped smoking during the first trimester, stopped smoking after the first trimester, smokes at most 5 cigarettes daily, smokes 6-10 cigarettes daily, smokes 11-20 cigarettes daily, smokes more than 20 cigarettes daily or smoker quantity unspecified). In the analysis smoking during pregnacy is treated as a binary.

[e] Parents were identified through the Civil Registration System and parental diagnoses obtained from the National Patient Register. Information an maternal infections during pregnancy was defined as a maternal diagnosis of infection nine month prior to the date of birth that corresponded to the gestational age of the child. Unknown parental history reflects individuals for which the father was unknown (N<80), the parent had died or left the country without a preceding diagnosis (N<10), or the parent id had been changed between the 2012 and 2016 data freezes (N<10). Of the 605 186 parental person years, only 111 264 occurred before Jan 1, 1969, where the recordings from PCRR starts [7], and of those only 30 800 person years (5%) occurred in individuals over age 10.

[f] All psychiatric hospital contacts with Schizophrenia as either main diagnosis (aktionsdiagnose/hoveddiagnose) or basic diagnosis (grundmorbus) in the Danish Psychiatric Central Research Register complete until December 31, 2016.

[g] Contains the total number of days admitted as 24 hour inpatient with a Schizophrenia diagnosis. For an admission date of the admission (unfinished admission) the admission is presumed ongoing until December 31, 2016. An exception for this rule is when the person has another contact in the Danish Psychiatric Central Research Register starting after the first unfinished admission (ptype = 0). In this case all unfinished admissions with ptype = 0 for this person are counted as having a duration of 1 day only. For persons with overlapping admissions with patient type 0 each day only counts once.

Table S 8: Result of the multinomial logistic regression of the association between k=5 clustering of the disease trajectories and schizophrenia risk factors and outcomes

| | Global assoc. test | | Cluster 1 | | Cluster 2 | | Cluster 3 | | Cluster 4 | |
|---|---|---|---|---|---|---|---|---|---|---|
| | LR | p-value | exp(beta) | p-value | exp(beta) | p-value | exp(beta) | p-value | exp(beta) | p-value |
| PGS - Education, Years | 12.4 | 1.46e-02 | 0.79 (0.68-0.93) | 3.42e-03 | 0.90 (0.80-1.01) | 7.28e-02 | 1.02 (0.88-1.17) | 0.80337 | 0.87 (0.75-1.00) | 5.76e-02 |
| Maternal Age | 21.2 | 2.94e-04 | 0.96 (0.94-0.99) | 7.63e-04 | 0.99 (0.97-1.00) | 1.17e-01 | 1.01 (0.99-1.03) | 0.30359 | 0.97 (0.96-1.00) | 1.39e-02 |
| Paternal Age | 13.9 | 7.61e-03 | 0.99 (0.97-1.01) | 2.94e-01 | 0.99 (0.98-1.00) | 9.71e-02 | 1.02 (1.00-1.03) | 0.03997 | 0.99 (0.98-1.01) | 3.97e-01 |
| Birth Length | 17.5 | 1.53e-03 | 0.95 (0.91-0.98) | 1.73e-03 | 0.98 (0.95-1.00) | 9.18e-02 | 1.01 (0.97-1.04) | 0.64274 | 0.95 (0.92-0.99) | 3.99e-03 |
| Birth Weight | 24.9 | 5.29e-05 | 0.57 (0.39-0.84) | 4.04e-03 | 0.91 (0.70-1.19) | 5.03e-01 | 1.59 (1.15-2.19) | 0.00460 | 0.89 (0.62-1.28) | 5.42e-01 |
| Maternal Smoking in Pregnancy | 12.5 | 1.42e-02 | 1.84 (1.25-2.71) | 2.09e-03 | 1.38 (1.01-1.90) | 4.65e-02 | 1.04 (0.67-1.62) | 0.85446 | 1.66 (1.01-2.72) | 4.40e-02 |
| Bacterial Infection | 44.8 | 4.46e-09 | 1.71 (1.38-2.13) | 8.88e-07 | 1.61 (1.38-1.89) | 2.73e-09 | 1.31 (1.08-1.60) | 0.00674 | 1.37 (1.12-1.68) | 2.14e-03 |
| Viral Infection | 15.8 | 3.35e-03 | 1.39 (1.09-1.77) | 7.93e-03 | 1.32 (1.10-1.58) | 3.05e-03 | 1.12 (0.89-1.41) | 0.34433 | 1.43 (1.14-1.80) | 1.83e-03 |
| Number of hospitalizations (SCZ) | 175.4 | 7.08e-37 | 3.20 (2.04-5.03) | 4.73e-07 | 6.44 (4.61-9.00) | 9.71e-28 | 1.66 (1.06-2.60) | 0.02511 | 7.36 (4.91-11.03) | 4.28e-22 |
| Total time hospitalized (SCZ) | 106.8 | 3.55e-22 | 1.16 (1.01-1.33) | 3.13e-02 | 1.44 (1.31-1.60) | 8.36e-13 | 1.09 (0.96-1.24) | 0.17819 | 1.75 (1.55-1.99) | 1.29e-18 |
| Maternal Diagnosis, Any Psychiatric | 40.9 | 2.77e-08 | 1.68 (1.36-2.08) | 1.87e-06 | 1.51 (1.28-1.77) | 5.88e-07 | 1.01 (0.82-1.25) | 0.92860 | 1.25 (1.02-1.53) | 3.32e-02 |
| Maternal Diagnosis, Schizophrenia | 10.6 | 3.10e-02 | 1.69 (1.02-2.82) | 4.33e-02 | 0.87 (0.55-1.36) | 5.34e-01 | 0.55 (0.28-1.06) | 0.07497 | 1.11 (0.66-1.86) | 6.96e-01 |
| Paternal Diagnosis, Any Psychiatric | 20.5 | 4.04e-04 | 1.41 (1.12-1.79) | 3.92e-03 | 1.40 (1.17-1.67) | 2.01e-04 | 1.29 (1.04-1.61) | 0.02138 | 1.46 (1.18-1.81) | 5.87e-04 |

Multinomial logistic regression is conducted using the cluster membership as dependent variable sequentially using the predictor variables as independent variable and treating Cluster 5 as reference. All regressions are adjusted for age and sex.
PGS - Educational attainment is adjusted additionally for 10 principal components and genotype wave.
Global assoc. test = four degree of freedom likelihood ratio (LR) test comparing full model to a model with all covariates, but without the predictor. p-values are unadjusted.

Table S 9: Associations the three principal MDS dimensions of psychiatric comorbidity trajectories and risk factors and outcomes in independent replication cohort

| | N | p.val |
|---|---|---|
| PGS - Education, Years | 630 | 0.00853 |
| Maternal Age | 856 | 0.00031 |
| Paternal Age | 843 | 0.15960 |
| Birth Length | 851 | 0.80479 |
| Birth Weight | 834 | 0.42617 |
| Maternal Smoking in Pregnancy | 459 | 0.00074 |
| Bacterial Infection | 870 | 0.23369 |
| Viral Infection | 870 | 0.16137 |
| Maternal Diagnosis, Any Psychiatric | 858 | 0.43808 |
| Maternal Diagnosis, Schizophrenia | 858 | 0.15617 |
| Paternal Diagnosis, Any Psychiatric | 858 | 0.88744 |
| Number of hospitalizations (SCZ) | 857 | 0.00021 |
| Total time hospitalized (SCZ) | 857 | 0.00043 |

MANCOVA performed with MDS dimension 1-3 as dependent variable and one degree of freedom and adjusting for age and sex. For genetic variables adjusting additionally for 10 principal components and genotype wave.p-values are unadjusted.

Table S 10: Linear regression of individual MDS dimension with significant association in main cohort conducted in the replication cohort

| | MDS dimension | $N_{replication}$ | $\beta_{main}$ | $\beta_{replication}$ |
|---|---|---|---|---|
| Maternal Smoking in Pregnancy | Dim1 | 459 | 0.713 | 0.112 |
| Bacterial Infection | Dim1 | 870 | 0.930 | 0.521 |
| Viral Infection | Dim1 | 870 | 0.541 | 0.506 |
| Maternal Diagnosis, Any Psychiatric | Dim1 | 858 | 0.846 | 0.307 |
| Paternal Diagnosis, Any Psychiatric | Dim1 | 858 | 0.544 | 0.146 |
| Maternal Diagnosis, Schizophrenia | Dim2 | 858 | 0.767 | -0.681 |
| PGS - Education, Years | Dim3 | 630 | -0.141 | -0.224 |
| Maternal Age | Dim3 | 856 | -0.029 | -0.040 |
| Paternal Age | Dim3 | 843 | -0.018 | -0.003 |
| Birth Length | Dim3 | 851 | -0.054 | -0.022 |
| Birth Weight | Dim3 | 834 | -0.372 | -0.282 |
| Number of hospitalizations (SCZ) | Dim3 | 857 | 1.706 | 0.118 |
| Total time hospitalized (SCZ) | Dim3 | 857 | 0.450 | 0.084 |

Linear regression was performed for the dimension with the strongest association in the main analysis. All regression were adjusted of age and sex.
For genetic variables adjusting additionally for 10 principal components and genotype wave.
This was done to test for sign concordance.

## Table S 11: Associations of significant variables across different parameter setting in 25-year subset

| | OM_jacc_.5 | OM_jacc_1 | HAM_jacc | OM_smc_.5 | OM_smc_1 | HAM_smc | OM_const_.5 | OM_const_1 | HAM_const | eucl_2 | chi2_2 | eucl_5 | chi2_5 | eucl_12 | chi2_12 | With imputation |
|---|---|---|---|---|---|---|---|---|---|---|---|---|---|---|---|---|
| PGS - Education, Years (N=1024) | 4.8e-01 | 4.8e-01 | 4.8e-01 | 1.0e-01 | 1.0e-01 | 1.0e-01 | 6.8e-01 | 7.9e-01 | 7.9e-01 | 9.0e-01 | 0.42 | 9.4e-01 | 0.123 | 5.8e-01 | 0.1300 | 1.6e-01 |
| Maternal Age (N=2584) | 1.1e-04 | 1.8e-04 | 1.8e-04 | 2.5e-04 | 2.5e-04 | 2.5e-04 | 2.8e-03 | 4.0e-03 | 4.1e-03 | 1.0e-02 | 0.43 | 1.2e-02 | 0.060 | 9.2e-03 | 0.0256 | 2.1e-06 |
| Paternal Age (N=2545) | 4.2e-02 | 5.5e-02 | 5.5e-02 | 5.3e-02 | 5.3e-02 | 5.3e-02 | 5.4e-02 | 9.6e-02 | 9.6e-02 | 2.3e-01 | 1.00 | 2.5e-01 | 0.865 | 1.1e-01 | 0.7579 | 1.8e-03 |
| Birth Length (N=3440) | 6.0e-04 | 7.8e-04 | 7.8e-04 | 6.1e-04 | 6.1e-04 | 6.1e-04 | 4.6e-03 | 6.2e-03 | 6.4e-03 | 4.5e-02 | 0.61 | 3.7e-02 | 0.142 | 2.9e-02 | 0.1144 | 6.4e-04 |
| Birth Weight (N=2551) | 7.1e-02 | 1.1e-01 | 1.1e-01 | 1.4e-01 | 1.4e-01 | 1.4e-01 | 1.6e-01 | 4.1e-01 | 4.1e-01 | 1.5e-01 | 0.17 | 1.1e-01 | 0.664 | 5.3e-02 | 0.5363 | 3.0e-02 |
| Bacterial Infection (N=3508) | 4.1e-11 | 8.4e-11 | 8.4e-11 | 2.8e-09 | 2.8e-09 | 2.8e-09 | 7.3e-09 | 3.0e-08 | 3.0e-08 | 1.5e-08 | 0.31 | 1.1e-08 | 0.850 | 8.6e-09 | 0.7072 | 4.3e-11 |
| Viral Infection (N=3508) | 2.8e-03 | 2.8e-03 | 2.8e-03 | 9.4e-03 | 9.4e-03 | 9.4e-03 | 3.9e-03 | 4.1e-03 | 4.1e-03 | 2.7e-03 | 0.87 | 2.6e-03 | 0.824 | 1.6e-03 | 0.4550 | 4.9e-03 |
| Maternal Diagnosis, Any Psychiatric (N=3451) | 3.0e-08 | 1.1e-08 | 1.1e-08 | 1.6e-09 | 1.6e-09 | 1.6e-09 | 1.4e-06 | 2.6e-07 | 2.6e-07 | 3.4e-07 | 0.54 | 4.8e-07 | 0.129 | 9.8e-07 | 0.0046 | 6.4e-10 |
| Maternal Diagnosis, Schizophrenia (N=3451) | 4.3e-03 | 2.6e-03 | 2.6e-03 | 1.2e-04 | 1.2e-04 | 1.2e-04 | 6.4e-02 | 4.2e-02 | 4.1e-02 | 1.5e-03 | 0.94 | 2.1e-03 | 0.427 | 1.5e-01 | 0.2047 | 3.3e-03 |
| Paternal Diagnosis, Any Psychiatric (N=3448) | 7.5e-05 | 6.0e-05 | 6.0e-05 | 3.5e-03 | 3.5e-03 | 3.5e-03 | 3.3e-04 | 8.8e-05 | 8.8e-05 | 5.6e-05 | 0.96 | 4.0e-05 | 0.879 | 9.0e-05 | 0.5567 | 1.2e-03 |
| Number of hospitalizations (SCZ) (N=3508) | 4.4e-19 | 4.4e-20 | 4.4e-20 | 8.1e-25 | 8.1e-25 | 8.1e-25 | 6.5e-12 | 5.8e-14 | 6.0e-14 | 5.0e-17 | 0.52 | 3.1e-17 | 0.073 | 3.5e-15 | 0.0415 | 5.9e-43 |
| Total time hospitalized (SCZ) (N=3508) | 2.7e-21 | 2.3e-22 | 2.3e-22 | 9.7e-14 | 9.7e-14 | 9.7e-14 | 1.0e-15 | 1.7e-18 | 1.8e-18 | 1.8e-20 | 0.62 | 6.9e-20 | 0.189 | 1.4e-19 | 0.1331 | 5.4e-26 |

OM = Optimal Matching, HAM= Hamming Distance,
Eucl = Euclidian distance (state distribition (number=k)),
chi2= chi-squared (state distribition(number=k)),
jacc = 1-jaccard coefficient, smc= Simple Matching coefficent,const= Constant Costs.
MANCOVAs done with first three dimensions of MDS as dependent variable, adjusting for age and sex.For genetic varibles adjustning additionally for 10 principal components and genotype wave.

Table S 12: Number of individuals with schizophrenia in the study and (expected) number of individuals with schizophrenia in the full population cohort

| | follow-up to Dec 31, 2012 | follow-up to Dec 31, 2016 |
|---|---|---|
| $N_{SCZ}$ in study sample | 5432 | 5432+870= 6302 |
| $N_{SCZ}$ in full population cohort (N=1 472 762) | 5432 | $5432 + 79 \times \frac{1472762}{30000} \approx 9300$ |

Here 79 is the number of individuals in the 30 000 random population sample assigned a schizophrenia diagnosis between December 31, 2012 and December 31, 2016.

## Supplementary References


[1] Peter C. Austin. “Balance diagnostics for comparing the distribution of baseline covariates between treatment groups in propensity-score matched samples”. In: *Statistics in Medicine* 28.25 (Nov. 2009), pp. 3083–3107. ISSN: 02776715. DOI: 10.1002/sim.3697. URL: http://doi.wiley.com/10.1002/sim.3697.

[2] J. C. Deville and G. Saporta. “Correspondence analysis, with an extension towards nominal time series”. In: *Journal of Econometrics* 22.1-2 (1983), pp. 169–189. ISSN: 03044076. DOI: 10.1016/0304-4076(83)90098-2.

[3] Alexis Gabadinho and Gilbert Ritschard. “Analyzing State Sequences with Probabilistic Suffix Trees : The PST R Package”. In: *Journal of Statistical Software* 72.3 (2016). DOI: 10.18637/jss.v072.i03.

[4] Brendan Halpin. “Multiple imputation for categorical time series”. In: *Stata Journal* 16.3 (2016), pp. 590–612. ISSN: 15368734. DOI: 10.1177/1536867x1601600303.

[5] Daniel Ho et al. “MatchIt: Nonparametric Preprocessing for Parametric Causal Inference”. In: *Journal of Statistical Software, Articles* 42.8 (2011), pp. 1–28. ISSN: 1548-7660. DOI: 10.18637/jss.v042.i08. URL: https://www.jstatsoft.org/v042/i08.

[6] Jan de Leeuw and Jacqueline Meulman. “A special Jackknife for Multidimensional Scaling”. In: *Journal of Classification* 3.1 (1986), pp. 97–112. ISSN: 01764268. DOI: 10.1007/BF01896814.

[7] O Mors, G P Perto, and P B Mortensen. “The Danish Psychiatric Central Research Register”. In: *Scand J Public Health* 39.7 Suppl (2011), pp. 54–57. ISSN: 1403-4948. DOI: 10.1177/1403494810395825. URL: http://www.ncbi.nlm.nih.gov/pubmed/21775352.

[8] Julie Nordgaard et al. “Variability in clinical diagnoses during the ICD-8 and ICD-10 era”. In: *Social Psychiatry and Psychiatric Epidemiology* 51.9 (2016), pp. 1293–1299. ISSN: 09337954. DOI: 10.1007/s00127-016-1265-9.

[9] C B Pedersen et al. “The iPSYCH2012 case – cohort sample : new directions for unravelling genetic and environmental architectures of severe mental disorders”. In: *Nature Publishing Group* 23.1 (2017), pp. 6–14. ISSN: 1359-4184. DOI: 10.1038/mp.2017.196. URL: http://dx.doi.org/10.1038/mp.2017.196.

[10] Carsten Bocker Pedersen et al. “A comprehensive nationwide study of the incidence rate and lifetime risk for treated mental disorders.” In: *JAMA psychiatry* 71.5 (2014), pp. 573–581. ISSN: 2168-6238 (Electronic). DOI: 10.1001/jamapsychiatry.2014.16.

[11] Matthias Studer and Gilbert Ritschard. “What matters in differences between life trajectories: A comparative review of sequence dissimilarity measures”. In: *Journal of the Royal Statistical Society. Series A: Statistics in Society* 179.2 (2016), pp. 481–511. ISSN: 1467985X. DOI: 10.1111/rssa.12125.

[12] Kristine Rømer Thomsen et al. “Changes in the composition of cannabis from 2000-2017 in Denmark: Analysis of confiscated samples of cannabis resin”. In: *Experimental and Clinical Psychopharmacology* 27.4 (2019), pp. 402–411. ISSN: 19362293. DOI: 10.1037/pha0000303.
